# Emergency management: microbial keratitis

**Published:** 2018-11-09

**Authors:** Simon Arunga, Matthew Burton

**Affiliations:** 1 Clinical Lecturer and Ophthalmologist: Mbarara University and Regional Referral Hospital Eye Centre, Mbarara, Uganda.; 2 Professor of International Eye Health: International Centre for Eye Health, London School of Hygiene & Tropical Medicine, London, UK.

**Microbial keratitis requires prompt diagnosis and immediate treatment to prevent severe loss of vision.**

Microbial keratitis is an infection of the cornea that can be caused by bacteria, fungi or protozoa such as *Acanthamoeba spp*. In low- and middle-income countries, management is often more challenging because of late presentation, the use of traditional eye medicines, insufficient diagnostic support, a lack of effective drugs and insufficient keratoplasty services.

Our experience in East Africa is that most patients will visit a primary health centre within a day or two of onset of symptoms, but may take another two weeks to reach the eye unit; by which time it can be too late to save the eye. All health care workers, including front-line primary health workers, must therefore know how to identify microbial keratitis early, provide immediate treatment, refer patients for specialist treatment and make sure they are able to take up the referral.

## Detecting microbial keratitis

The clinical presentation of microbial keratitis has been covered in detail in previous editions of this journal.^1^,^2^

Patients usually present with reduced vision, pain, discharge, and red eyes. They may have a history of trauma and traditional eye medicine use.

Measure visual acuity first and record it, then examine the eye to look for signs of microbial keratitis.

## Equipment

- A torch with a bright light, a direct ophthalmoscope, or the Arclight
- Fluorescein strips
- A blue light source. You can use a simple blue-coloured filter (a thin plastic sheet) on your light source
- Magnifying loupes or a simple pair of reading glasses can be helpful for seeing finer details, such as a corneal foreign body.

## Signs

**Figure 1:**
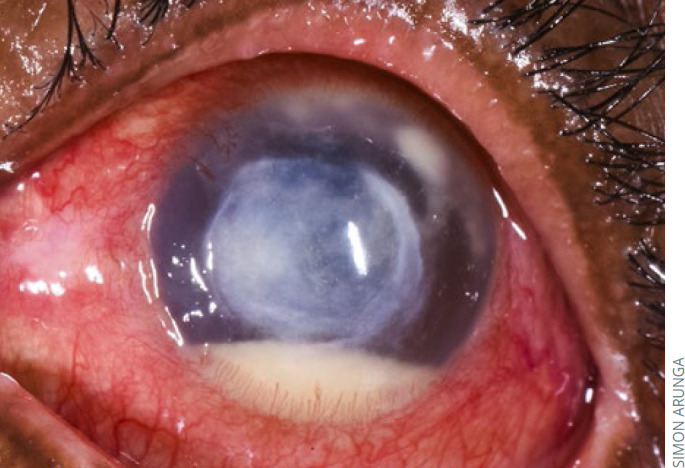
Microbial keratitis with ciliary injection, corneal infiltrate and a hypopyon. This patient also has satellite lesions superiorly

**Figure 2:**
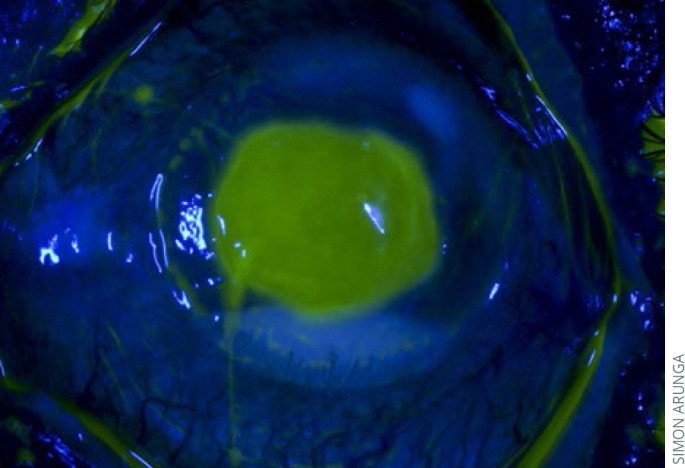
Microbial keratitis. The corneal epithelial defect shows up green with fluorescein staining

Look for signs of:

- Ciliary injection: a red eye that involves the branches of the anterior ciliary arteries
- Corneal infiltrate: creamy white material in the cornea
- A hypopyon: creamy white material that has collected at the bottom of the iris (Figure 1)
- A corneal epithelial defect which shows up as green with fluorescein staining (Figure 2).

Write down and draw all your observations, including the size, shape and location of any lesions. The patient in Figures 1 and 2 has advanced microbial keratitis.

## Initial management

**Figure 3:**
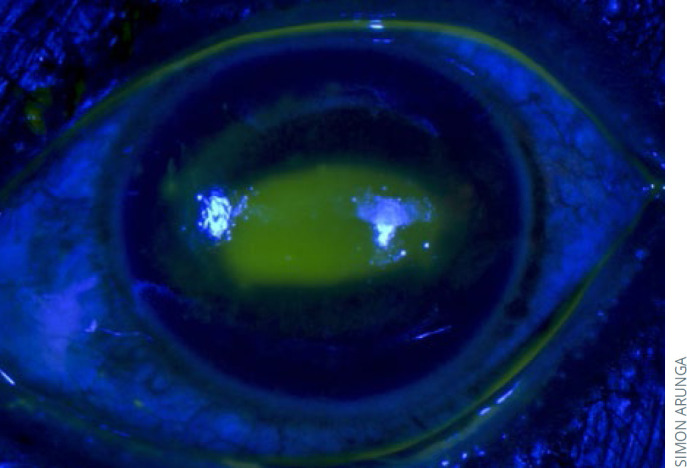
Fluorescein staining confirms the presence of a corneal abrasion

A **corneal abrasion** (Figure 3) may develop into microbial keratitis. At primary level, give chloramphenicol eye ointment 3 times a day for 3 days. Look for a corneal foreign body and refer if present. Treat as microbial keratitis if it has not resolved after 3 days.

Refer patients urgently if you suspect **microbial keratitis**. Prescribe broad-spectrum antibiotic eye drops with instructions to instil them hourly, day and night (as long as the patient is awake), until they are seen at the referral centre. Microbial keratitis should be managed in a setting where full microbiology investigation and clinical assessment can be performed. Management of microbial keratitis, including the preparation of fortified antibiotics, has been described in this journal previously.^1^,^3^

## The referral process

Referral is not simple for the patient. Many live in rural areas and referral means travelling long distances to a large urban eye unit that they have never been to before, incurring considerable expenses. These are barriers to the referral process and need to be considered.

In your referral letter, document your findings, including baseline measurements, initial treatment and reason for referral, and then contact the referral centre about the patient. Prior notice also helps the referral centre to prepare the necessary items such as agar plates.

Patients must feel supported and need to know that someone will be expecting them. This might mean giving them a phone number to call when they arrive.

Explain the purpose and urgency of the referral in order to ensure that the patient attends. Microbial keratitis, especially fungal infections, tend to resolve slowly, and counselling helps to manage patients' expectations and keep them hopeful.

## How you can be prepared

- Equip your health facility with a torch, fluorescein strips and broad-spectrum antibiotics
- Ensure that the details of the nearest referral centre are clearly written down, where everyone can see it
- Ensure that everyone in the team knows how to prepare broad-spectrum antibiotics and has access to printed instructions for doing so
- Print out a decision-making guide and referral checklist and display these in your clinic.

**Referral checklist**

- Baseline examination, including visual acuity, done and recorded
- Initial treatment started and documented in detail
- Reason for referral documented clearly and communicated to the patient
- Patient understands the expected healing prognosis and timeframe
- Referral centre contacted
- Patient knows when the referral centre is open and is given clear directions
- Patient is given the phone number of the coordinator at the referral centre to call once they arrive so that they are seen urgently.
